# The +3187A/G *HLA-G* polymorphic site is associated with polar forms and reactive reaction in leprosy

**DOI:** 10.1002/mgg3.14

**Published:** 2013-06-07

**Authors:** N Lucena-Silva, M A G Teixeira, A de L Ramos, R S de Albuquerque, G T N Diniz, C T Mendes-Junior, E C Castelli, E A Donadi

**Affiliations:** 1 Departamento de Imunologia, Centro de Pesquisas Aggeu Magalhães, Fundação Oswaldo Cruz50670-420, Recife-PE, Brazil; 2 Laboratório de Biologia Molecular, Departamento de Oncologia Pediátrica, Hospital IMIP50070-550, Recife-PE, Brazil; 3 Divisão de Imunologia Clínica, Departamento de Medicina, Escola de Medicina de Ribeirão Preto, Universidade de São Paulo14040-900, Ribeirão Preto-SP, Brazil; 4 Departamento de Dermatologia da Universidade de Pernambuco e Centro Integrado de Saúde Amaury de Medeiros, Secretaria de Saúde de Pernambuco52030-010, Recife-PE, Brazil; 5 Laboratorio de Métodos Quantitativos, Centro de Pesquisas Aggeu Magalhães, Fundação Oswaldo Cruz50670-420, Recife-PE, Brazil; 6 Departamento de Química, Faculdade de Filosofia, Ciências e Letras de Ribeirão Preto, Universidade de São Paulo14040-901, Ribeirão Preto-SP, Brazil; 7 Departamento de Biologia Geral, Instituto de Ciências Biológicas, Universidade Federal de Goias – UFG74001-970, Goiânia-GO, Brazil

**Keywords:** 3′ UTR polymorphism, Hansenic reaction, HLA-G, leprosy

## Abstract

Considering that variability in immune response genes has been associated with susceptibility to leprosy and with disease severity, leprosy presents clinicopathological variants that are highly associated with the immune response, HLA-G has a well-recognized role in the modulation of the immune response, and polymorphisms at the 3′ untranslated region (UTR) of the *HLA-G* gene may influence HLA-G production, we studied the polymorphic sites at the 3′ UTR of the *HLA-G* gene in leprosy and their association with disease severity. We evaluated by sequencing analysis the allele, genotype, and haplotype frequencies of the 3′ UTR *HLA-G* polymorphic sites (14-bpINDEL/+3003C-T/+3010C-G/+3027A-C/+3035C-T/+3142C-G/+3187A-G/+3196C-G) in 146 individuals presenting reactive leprosy from a highly endemic area, and associated with bacillary load and the type of reactive leprosy. A total of 128 healthy subjects were also studied. Allele, genotype, and haplotype frequencies for the 3′ UTR *HLA-G* polymorphisms in leprosy patients did not differ from those observed in healthy donors. The +3187A allele was responsible for protection against the development of multibacillary leprosy in a dominant model (AA + AG)/GG, OR = 0.11, *P* = 0.018), and the +3187A allele and +3187A-A genotype were overrepresented in type II reactive leprosy reaction. The effect of genetic factors on leprosy susceptibility may be hidden by environmental components in highly endemic areas. The *HLA-G* + 3187A polymorphic site, which is related to unstable mRNA production, was associated with the development of polar forms of leprosy and reactive leprosy reaction.

## Introduction

Leprosy is a complex infectious disease caused by exposure to *Mycobacterium leprae* (*M. leprae*) that occurs as a consequence of determinants of microbial pathogenicity and interactions of host genetic-environmental factors.

Five main clinical forms of leprosy have been identified based on histopathological, clinical, and bacteriological features, which correlate with the immune response. At one pole of the clinical spectrum is tuberculoid–tuberculoid leprosy (TT), presenting few hypopigmented anesthetic skin lesions, and undetectable bacillary load, inducing a typical Th1 cell-mediated immune response, formation of granulomas, and bacillus arresting. At the other pole is lepromatous leprosy (LL) showing numerous skin lesions with high bacillary load, inducing Th2 cell-mediated immune response, which does not promote the formation of granulomas, but favors bacillary replication and disease progression. Between them there are three borderline leprosy forms, named borderline–tuberculoid (BT), borderline–borderline (BB), and borderline–lepromatous (BL). There is also an intermediate state with an unstable immune response, and an earlier undefined state named indeterminate leprosy (de Oca EPM 2011; Sampaio et al., 2012).

WHO operational classification defines leprosy as paucibacillary (PB) and multibacillary (MB) forms, according to the presence of up to five or six or more skin lesions, respectively, independently of microbiological examination. This approach facilitates the establishment of the appropriate treatment and predicts complications. Roughly, PB leprosy corresponds to the TT, BT, and indeterminate clinical forms, and MB leprosy to the LL, BL, and BB forms (de Oca EPM 2011). However, it is possible that a fraction of cases with BL present less than five skin lesions, being bacilloscopy positive (Cavalcanti et al. 2012).

Leprosy reaction is an exacerbated immunological response due to the presence of circulating mycobacterial antigens. Type I leprosy (reversal) reaction is more frequently observed in BL due to the activation of the cell-mediated immunity, which drives the immune response to the tuberculoid pole, and its characteristic features are edema, erythema or ulceration of skin lesions and neuritis (Cuevas et al. 2007). Type II reaction occurs in 40–50% of LL (Moraes et al. 2001), and the most common clinical variant is erythema nodosum leprosum (ENL), which is characterized by necrotic lesions in the epidermis and dermis, and painful nerve swelling associated with systemic inflammatory symptoms. The pathogenesis of ENL is related to the abundant circulating histiocytes full of bacilli, in the presence of a poor T cell-mediated response to antimycobacterial drug treatment, releasing mycobacteria antigens in the blood circulation. Specific antibodies and complement molecules recognize and bind to these circulating antigens, leading to the formation of medium-sized immune complexes that precipitate in blood vessel walls, recruiting neutrophils and favoring vessel wall necrosis (Laal et al. 1985; Cuevas et al. 2007).

Accumulating evidence indicates that genetic variability at gene loci responsible for the immune response may play an important role in determining individual susceptibility to leprosy (de Oca EPM 2011). Polymorphisms in innate immune receptor, *mannose-binding lectin* (*MBL*), *vitamin D receptor* (*VDR*), and *nucleotide-binding oligomerization domain* (*NOD2*) genes influence the immune response and the differentiation of the clinical phenotypes of leprosy (Roy et al. 1999; de Messias-Reason et al. 2007; Sapkota et al. 2010; Berrington et al. 2010; Vasconcelos et al. 2011). Two other genes involved in the innate immunity response, the *NRAMP-1* gene, which is responsible for the intracellular survival of the bacilli, and the human *B-defensin* 1 gene that codes for an antimicrobial effector molecule, were also associated with susceptibility to leprosy and to disease severity (Gruenheid and Gros 2000; de Oca et al. 2009).

The complex genetic regulation of the adaptive immune response resulting from the balance between different secreted cytokines is crucial for the outcome of the host-pathogen interactions, that is, elimination of the bacilli, development of the resistant form of leprosy (TT), or the more severe form (LL). This has been confirmed by the observation that polymorphisms of key cytokines genes, such as TNF (Santos et al. 2002; Sapkota et al., 2010; Cardoso et al. 2011), IL-12 (Alvarado-Navarro et al. 2008), IL-4 (Yang et al. 2011; Sampaio et al. 2012), IL-10, TGF-B, IL-6, and their respective receptors (Aggarwal et al. 2011) have been associated with susceptibility to and severity of leprosy.

HLA-related gene polymorphisms have also been investigated in adaptive immunity against leprosy. HLA class I and class II alleles were associated with susceptibility to and severity of leprosy (Shaw et al. 2001; Shankarkumar et al. 2003). However, nothing is known about the role of nonclassical HLA class I molecules that are involved in the regulation of the immune response. In this context, the HLA-G molecule has a well-recognized role in the downregulation of the immune response, limiting inflammation by binding to several leukocyte inhibitory receptors, including KIR2DL4 (in NK and T cells), ILT2 (in NK, APC, and T cells), ILT4 (in myeloid cells), and CD8 (in T cells) receptors (Donadi et al. 2011). The major effects of HLA-G expression include: (i) inhibition of cytolysis mediated by NK, NKT, and CTL lymphocytes; (ii) suppression of CD4+ T-cell proliferation; (iii) induction of T-cell anergy; (iv) generation of suppressor regulatory cells; (v) induction of CD8+ T-cell apoptosis by soluble HLA-G isoforms; (vi) IL-10 upregulation; (vii) shift to Th2 immune response polarization (Carosella et al. 2008; Huang et al. 2009; Donadi et al. 2011). Polymorphic sites along the *HLA-G* gene have been described and associated with diverse patterns of HLA-G in many diseases (Larsen et al. 2010; Castelli et al. 2011; Ferguson et al. 2012). Considering that: (i) HLA-G regulation includes stimulation of IL-10 expression, (ii) IL-10 is a key cytokine of Th2 cell-mediated immunity, associated with the pathogenesis of the severe forms of leprosy, (iii) HLA-G expression may be influenced by 3′ untranslated region (3′ UTR) variation sites by posttranscriptional mechanisms, in which many polymorphic sites have already been associated with the magnitude of HLA-G production, we aimed to study the polymorphic sites at the 3′ UTR of the *HLA-G* gene in leprosy and the possible association of polymorphisms with disease severity.

## Patients and Methods

### Population

A total of 146 individuals exhibiting reactional leprosy (76 with positive bacilloscopy and 70 with negative baciloscopy at diagnosis) were enrolled from those attending the Dermatology and Leprosy Outpatient Care at the Centro Integrado de Saúde Amaury de Medeiros, University of Pernambuco, Recife, Northeast of Brazil. Patients were also grouped according to the type of reactive reaction they presented: Type I or reverse reaction and Type II or erythema nodosum leprae. Fifteen patients who presented episodes of both types of reactional leprosy were not considered in this particular analysis. The association of the polymorphisms of the 3′ UTR of the *HLA-G* gene with leprosy susceptibility was assessed by comparison of data regarding the *HLA-G* 3′ UTR polymorphisms of 128 healthy unrelated blood donors from the same geographical region as the patients, whose 3′ UTR evaluation was previously published (Lucena-Silva et al. 2012). The proportion of healthy individuals exhibiting European (25%), Admixture (69%), and African (6%) background was closely similar to patients (28%, 62%, 10%, respectively, *P* = 0.445). The proportion between men and women in health population (86%:14%) differed from that observed in patients (65%:35%; *P* = 0.0001). Differences on frequencies of alleles, genotypes, and haplotypes were investigated according to the bacillus burden and type of reactive reaction to evaluate the possible role of genetic polymorphism on disease severity.

The study protocol was approved by the Ethics Committee of the Centro de Pesquisas Aggeu Magalhães (protocol # CAAE 0040.0.095.000-06) and Centro Integrado de Saúde Amaury de Medeiros (protocol # CAAE 0009.0.250.250-07), and written informed consent was obtained from all participants.

### Amplification and sequencing of the 3′ UTR of the HLA-G gene

Genomic DNA was extracted from peripheral blood mononuclear cells using the DNAzol reagent (Invitrogen, Carlsbad, CA) according to manufacturer's instructions. Amplification of the *HLA-G* 3′ UTR between positions +2945 and +3259 was performed as described elsewhere (Castelli et al. 2010). The amplification product was first evaluated using 2% agarose gel. PCR products containing the amplified fragment of approximately 350 bp were directly sequenced. Homozygous individuals for the presence or absence of the 14-bp fragment were sequenced in both directions using the Big Dye Terminator kit and the ABI3100 Genetic Analyzer (Applied Biosystems ®, Foster City, CA). Heterozygous individuals of 14 bp were sequenced only with the reverse primer. All polymorphic sites found in the *HLA-G* 3′ UTR region were individually annotated.

### Statistical analysis

The allelic and genotypic frequencies were estimated by direct counting. Adherence of genotypic proportions to Hardy–Weinberg equilibrium (HWE) expectations and Linkage disequilibrium (LD) between each pair of loci were evaluated using the ARLEQUIN program version 3.5.1.2 (Excoffier et al. 2005).

The most likely haplotype pair for each sample was determined by two independent computational methods without taking into account any prior information. The PHASE method (Stephens et al. 2001) was applied using the PHASE program version 2.1. The expectation-maximization (EM) algorithm (Excoffier and Slatkin 1995) was also used to estimate haplotype frequencies simultaneously in patient and control groups using the ARLEQUIN program. Haplotypes inferences using the EM and Phase methods with probabilities of at least 0.99 were considered for further analysis.

To compare the allele, genotype, and haplotype frequencies between patients and controls and between selected group of patients, we used the two-tailed Fisher exact test, and the *P*-value was considered to be statistically significant when <0.05. The relative risk was estimated by calculating the odds ratio (OR) with the 95% confidence interval (95% CI). Analyses of contingency tables were performed using GraphPad Prism version 5.01 for Windows (GraphPad Software, San Diego, CA, http://www.graphpad.com).

## Results

The demographic, clinical, and main laboratory features of the 146 leprosy individuals studied are shown in Table 1. The proportion of MB/PB individuals was similar (76:71); however, the proportion of those who presented type I reaction (102 cases) was threefold higher than the proportion of those with type II (29 cases).

**Table 1:** Features of individuals with reactional leprosy according the baciloscopy at diagnosis and the type of reactive state

Features	Multi (*n* = 76)	Pauci (*n* = 70)	Type I (*n* = 102)	Type II (*n* = 29)
Age				
Range	12–84	13–86	12–86	20–84
Median	41	43	43	36
Gender				
Male	53	42	66	20
Female	23	28	36	9
Ancestry				
European	15	26	33	5
Admixture	55	36	57	22
African	6	8	12	2
BAAR at diagnosis				
Positive	76	0	35	26
Negative	0	70	67	3
Clinical forms				
Lepromatous (LL)	31	0	6	18
Borderline (BL)	45	45	72	10
Tuberculoid (TT)	0	25	24	1
Reactional leprosy				
Type I	35	67	102	0
Type II	26	3	0	29
Type I and Type II*	15	0	–	–
Onset of the reactive episode				
During treatment	10	6	9	4
< 6 months of end treatment	65	58	89	25
> 6 months of end treatment	1	6	7	0

* Individuals who presented both type I and Type II reactional leprosy were not considered for the analysis on reactional state.

All previously described polymorphic sites, including the 14-bp INS/DEL (GenBank ID: rs1704), +3003C/T (GenBank ID: rs1707), +3010C/G (GenBank ID: rs1710), +3027A/C (GenBank ID: rs17179101), +3035C/T (GenBank ID: rs17179108), +3142C/G (GenBank ID: rs1063320), +3187A/G (GenBank ID: rs9380142), and +3196C/G (GenBank ID: rs1610696) were observed. Strong LD was observed between each possible pair among the polymorphic sites studied, except between +3003C/T and +3035C/T (*P* = 0.341). The observed heterozygosis for all genotypes did not differ from the expected one, showing that the genotype distribution fit HWE.

The frequencies of alleles, genotypes, and haplotypes of the *HLA-G* 3'UTR polymorphic sites did not differ between leprosy individuals and healthy blood donors from the same geographic region (Table 2). After stratification of the patients according to bacillary load at diagnosis into MB and PB groups, we found that the frequency of the +3187 G-G genotype was associated with MB leprosy (OR = 9.27, *P* = 0.018). The frequency of the +3187 G allele that occurs only in the UTR-1 haplotype was closely similar among MB (28%) and PB (21%) leprosy variants (*P* = 0.22). The +3187 G allele was more frequently observed in heterozygosis in PB leprosy. These findings are compatible with a recessive model (AA + AG)/GG, OR = 0.11, *P* = 0.018), which is responsible for protection against the development of MB leprosy, as observed in Table 2.

**Table 2:** Frequencies of alleles, genotypes, and haplotypes of the 3′ untranslated region of the HLA-G gene polymorphic sites in individuals with reactional leprosy according the baciloscopy at diagnosis

Polymorphism	Multi (*n* = 76)	Pauci (*n* = 70)	*P*-value	OR (IC 95%)	All (*n* = 146)	Healthy (*n* = 128)	*P*-value	OR (IC 95%)
14-bp INS	0.474	0.400	0.238	1.35 (0.85–2.15)	0.438	0.375	0.140	1.30 (0.92–1.83)
14-bp DEL	0.526	0.600		0.74 (0.47–1.18)	0.562	0.625		0.77 (0.55–1.08)
14-bp INS/14-bp INS	0.237	0.129	0.135	2.10 (0.87–5.06)	0.185	0.156	0.630	1.23 (0.65–2.31)
14-bp INS/14-bp DEL	0.474	0.543	0.413	0.76 (0.39–1.45)	0.507	0.438	0.276	1.32 (0.82–2.13)
14-bp DEL/14-bp DEL	0.289	0.329	0.720	0.83 (0.41–1.68)	0.308	0.406	0.101	0.65 (0.40–1.07)
+3003C	0.079	0.136	0.131	0.55 (0.25–1.17)	0.106	0.066	0.129	1.67 (0.90–3.10)
+3003T	0.921	0.864		1.83 (0.85–3.93)	0.894	0.934		0.60 (0.32–1.11)
+3003C/+3003C	0.000	0.000			0.000	0.000		
+3003C/+3003T	0.158	0.271	0.108	0.50 (0.22–1.13)	0.212	0.133	0.111	1.76 (0.92–3.36)
+3003T/+3003T	0.842	0.729	0.108	1.99 (0.88–4.47)	0.788	0.867		0.57 (0.30–1.09)
+3010C	0.579	0.571	0.906	1.03 (0.65–1.64)	0.575	0.539	0.438	1.12 (0.83–1.62)
+3010G	0.421	0.429		0.97 (0.61–1.54)	0.425	0.461		0.86 (0.62–1.21)
+3010C/+3010C	0.382	0.314	0.487	1.35 (0.68–2.67)	0.349	0.289	0.302	1.32 (0.79–2.20)
+3010C/+3010G	0.395	0.514	0.183	0.62 (0.32–1.19)	0.452	0.500	0.468	0.83 (0.51–1.33)
+3010G/+3010G	0.224	0.171	0.534	1.39 (0.61–3.17)	0.199	0.211	0.881	0.93 (0.52–1.67)
+3027A	0.066	0.043	0.448	1.57 (0.56–4.45)	0.055	0.035	0.310	1.59 (0.69–3.67)
+3027C	0.934	0.957		0.63 (0.22–1.80)	0.945	0.965		0.63 (0.27–1.45)
+3027A/+3027A	0.013	0.000	1.000	2.80 (0.11–69.96)	0.007	0.008	1.000	0.88 (0.05–14.16)
+3027A/+3027C	0.105	0.086	0.783	1.26 (0.41–3.82)	0.096	0.055	0.257	1.83 (0.20–4.70)
+3027C/+3027C	0.882	0.914	0.592	0.70 (0.24–2.07)	0.897	0.938	0.278	0.58 (0.24–1.42)
+3035C	0.862	0.857	1.000	1.04 (0.54–2.01)	0.860	0.871	0.709	0.91 (0.55–1.48)
+3035T	0.138	0.143	1.000	0.96 (0.50–1.86)	0.140	0.129		1.10 (0.67–1.81)
+3035C/+3035C	0.750	0.743	1.000	1.04 (0.49–2.19)	0.747	0.766	0.779	0.90 (0.52–1.57)
+3035C/+3035T	0.224	0.229	1.000	0.97 (0.45–2.11)	0.226	0.211	0.772	1.09 (0.61–1.94)
+3035T/+3035T	0.026	0.029	1.000	0.92 (0.13–6.71)	0.027	0.023	0.730	1.44 (0.34–6.14)
+3142C	0.408	0.414	1.000	0.97 (0.61–1.55)	0.411	0.449	0.388	0.86 (0.61–1.20)
+3142G	0.592	0.586		1.03 (0.64–1.64)	0.589	0.551		1.17 (0.83–1.64)
+3142C/+3142C	0.197	0.143	0.510	1.48 (0.61–3.54)	0.171	0.203	0.536	0.81 (0.44–1.49)
+3142C/+3142G	0.421	0.543	0.185	0.61 (0.32–1.18)	0.479	0.492	0.904	0.95 (0.59–1.53)
+3142G/+3142G	0.382	0.314	0.487	1.35 (0.68–2.67)	0.349	0.305	0.443	1.23 (0.74–2.04)
+3187A	0.717	0.786	0.223	0.69 (0.40–1.18)	0.750	0.701	0.211	1.28 (0.88–1.87)
+3187G	0.283	0.214		1.45 (0.85–2.47)	0.250	0.299		0.78 (0.54–1.14)
+3187A/+3187A	0.553	0.586	0.739	0.87 (0.45–2.68)	0.568	0.496	0.274	1.34 (0.83–2.16)
+3187A/+3187G	0.329	0.400	0.394	0.74 (0.37–1.45)	0.363	0.409	0.456	0.82 (0.50–1.34)
+3187G/+3187G	0.118	0.014	**0.018**	**9.27 (1.14–75.21)**	0.068	0.094	0.506	0.70 (0.29–1.69)
+3196C	0.684	0.757	0.193	0.70 (0.41–1.16)	0.719	0.764	0.242	0.79 (0.54–1.17)
+3196G	0.316	0.243		1.44 (0.86–2.41)	0.281	0.236		1.26 (0.86–1.86)
+3196C/+3196C	0.487	0.557	0.412	0.75 (0.39–1.45)	0.521	0.606	0.179	0.71 (0.44–1.14)
+3196C/+3196G	0.395	0.400	1.000	0.98 (0.50–1.90)	0.397	0.315	0.166	1.43 (0.87–2.36)
+3196G/+3196G	0.118	0.043	0.133	3.00 (0.78–11.57)	0.082	0.079	1.000	1.05 (0.44–2.51)
Haplotypes								
UTR-1 (DTGCCCGC)	0.283	0.214	0.223	1.45 (0.85–2.47)	0.250	0.295	0.248	0.80 (0.54–1.16)
UTR-2 (ITCCCGAG)	0.303	0.229	0.186	1.47 (0.87–2.48)	0.267	0.228	0.322	1.23 (0.83–1.82)
UTR-3 (DTCCCGAC)	0.118	0.186	0.140	0.59 (0.31–1.13)	0.151	0.157	0.905	0.95 (0.60–1.51)
UTR-4 (DCGCCCAC)	0.079	0.136	0.129	0.54 (0.25–1.15)	0.106	0.067	0.130	1.66 (0.89–3.07)
UTR-5 (ITCCTGAC)	0.072	0.100	0.412	0.70 (0.31–1.60)	0.086	0.087	1.000	0.99 (0.54–1.80)
UTR-6 (DTGCCCAC)	0.046	0.064	0.609	0.70 (0.25–1.94)	0.055	0.087	0.177	0.61 (0.31–1.19)
UTR-7 (ITCATGAC)	0.066	0.043	0.448	1.57 (0.56–4.45)	0.055	0.035	0.311	1.58 (0.68–3.64)
UTR-8 (ITGCCGAG)	0.013	0.014	1.000	0.92 (0.13–6.62)	0.014			
UTR-15 (ITCCCGAC)	0.020	0.014	1.000	1.39 (0.23–8.44)	0.017	0.024	0.762	0.72 (0.22–2.39)
Others						0.020		

Bold values denote differences were considered statistically significant with *P*-value <0.05.

We further evaluated the association between the 3′ UTR *HLA-G* polymorphisms and the type of reactive reaction presented by the patients. In this analysis, patients exhibiting both types of reaction (15 cases) were not considered. The +3187 A allele (*P* = 0.015) and +3187 A-A genotype (*P* = 0.032) were overrepresented in individuals who showed type II reaction, while the +3187 G allele was overrepresented in those who showed type I reaction (*P* = 0.015). Leprosy patient carrying the UTR-1 haplotype had a 2.8 higher risk to present type I leprosy than patients with type II (*P* = 0.015) (Table 3).

**Table 3:** Frequencies of alleles, genotypes, and haplotypes of polymorphic sites on the 3′ untranslated region of the HLA-G gene in individuals with reactional leprosy according the type of reactive reaction

Polymorphism	Type I (*n* = 102)	Type II (*n* = 29)	*P*-value	OR (IC 95%)
14-bp INS	0.426	0.500	0.369	0.74 (0.41–1.34)
14-bp DEL	0.574	0.500		1.35 (0.75–2.41)
14-bp INS/14-bp INS	0.167	0.241	0.416	0.63 (0.23–1.70)
14-bp INS/14-bp DEL	0.520	0.517	0.238	0.50 (0.19–1.34)
14-bp DEL/14-bp DEL	0.314	0.241	0.500	1.44 (0.56–3.71)
+3003C	0.098	0.138	0.469	0.68 (0.28–1.63)
+3003T	0.902	0.862		1.47 (0.61–3.54)
+3003C/+3003C	0.000	0.000		
+3003C/+3003T	0.196	0.276	0.441	0.64 (0.25–1.66)
+3003T/+3003T	0.804	0.724	0.411	1.56 (0.60–4.04)
+3010C	0.564	0.638	0.367	0.73 (0.40–1.34)
+3010G	0.436	0.362	0.367	1.36 (0.75–2.49)
+3010C/+3010C	0.314	0.448	0.191	0.56 (0.24–1.31)
+3010C/+3010G	0.500	0.379	0.295	1.64 (0.70–3.81)
+3010G/+3010G	0.186	0.172	1.000	1.10 (0.37–3.25)
+3027A	0.059	0.052	1.000	1.15 (0.31–4.21)
+3027C	0.941	0.948		0.87 (0.24–3.20)
+3027A/+3027A	0.010	0.000		
+3027A/+3027C	0.098	0.103	1.000	0.94 (0.24–3.68)
+3027C/+3027C	0.892	0.897	1.000	0.95 (0.25–3.68)
+3035C	0.848	0.862	1.000	0.89 (0.39–2.07)
+3035T	0.152	0.138		1.12 (0.48–2.59)
+3035C/+3035C	0.725	0.724	1.000	1.01 (0.40–2.54)
+3035C/+3035T	0.245	0.276	0.809	0.85 (0.34–2.16)
+3035T/+3035T	0.029	0.000	1.000	2.08 (0.10–41.36)
+3142C	0.426	0.328	0.225	1.53 (0.83–2.82)
+3142G	0.574	0.672		0.66 (0.35–1.21)
+3142C/+3142C	0.167	0.103	0.562	1.73 (0.47–6.39)
+3142C/+3142G	0.520	0.448	0.534	1.33 (0.58–3.05)
+3142G/+3142G	0.314	0.448	0.191	0.56 (0.24–1.31)
+3187A	0.725	0.879	**0.015**	0.36 (0.16–0.85)
+3187G	0.275	0.121		2.76 (1.18–6.44)
+3187A/+3187A	0.520	0.759	**0.032**	0.34 (0.14–0.88)
+3187A/+3187G	0.412	0.241	0.128	2.20 (0.86–5.62)
+3187G/+3187G	0.069	0.000	0.347	4.63 (0.26–83.63)
+3196C	0.745	0.655	0.185	1.54 (0.82–2.88)
+3196G	0.255	0.345		0.65 (0.34–1.22)
+3196C/+3196C	0.549	0.448	0.401	1.50 (0.65–3.43)
+3196C/+3196G	0.392	0.414	0.833	0.91 (0.39–2.12)
+3196G/+3196G	0.059	0.138	0.227	0.39 (0.10–1.49)
Haplotypes				
UTR-1 (DTGCCCGC)	0.277	0.121	**0.015**	2.80 (1.20–6.53)
UTR-2 (ITCCCGAG)	0.248	0.310	0.397	0.73 (0.38–1.39)
UTR-3 (DTCCCGAC)	0.144	0.172	0.676	0.80 (0.37–1.77)
UTR-4 (DCGCCCAC)	0.094	0.138	0.335	0.65 (0.27–1.57)
UTR-5 (ITCCTGAC)	0.094	0.086	1.000	1.10 (0.39–3.09)
UTR-6 (DTGCCCAC)	0.054	0.069	0.749	0.78 (0.24–2.54)
UTR-7 (ITCATGAC)	0.059	0.052	1.000	1.16 (0.32–4.25)
UTR-8 (ITGCCGAG)	0.010	0.034	0.216	0.28 (0.04–2.03)
UTR-15 (ITCCCGAC)	0.020	0.017	1.000	1.15 (0.13–10.51)

Bold values denote differences were considered statistically significant with *P*-value <0.05.

## Discussion

We reported that the allele and genotype frequencies of eight well-characterized polymorphic sites at the 3′ UTR of the *HLA-G* gene did not differ between leprosy patients and healthy blood donors from Pernambuco State, Northeastern Brazil. The prevalence of leprosy in Pernambuco State is 31.78 per 100,000 inhabitants, and the coefficient of detection of new cases in younger individuals aged less than 15 years is 12.97, which represents more than twice the national coefficient of 5.39 per 100,000 in youngsters (Bulletin of Brazilian Health Ministry 2010). In this scenario of high transmission and high prevalence rate, the contribution of genetic factors to the development of leprosy might not be identified, and the disease risk may be a result of the joint effect of genotype and environmental exposure (Yang and Khoury 1997). In this context, we have previously evaluated genetic variants in two polymorphic genes highly implicated in the immune response against *M. leprae,* but no association was found. We have shown that *N-RAMP* (Teixeira et al. 2010) and *MBL* (Vasconcelos et al. 2011) gene polymorphisms were not associated with susceptibility to leprosy in the same study population. Taken together, these findings indicate that environmental overexposure to infectious agents may hide the participation of genetic factors in these diseases. Further study of genetic epidemiology should be performed to measure the contribution of genetic factors in the development of leprosy.

Considering only leprosy patients, we found an association of the +3187A/G polymorphism with severe disease and reactive leprosy reaction. The +3187 A/G polymorphic site is located in the vicinity of a recognized Adenine–Uridine rich motif that mediates *HLA-G* mRNA degradation. Indeed, the presence of an Adenine at position +3187 is associated with decreased mRNA stability in vitro and low HLA-G production by the placenta during pregnancy, favoring the development of preeclampsia (Yie et al. 2008).

HLA-G-expressing CD4^+^ T regulatory cells have been reported in peripheral blood of healthy individuals, exerting their activity of balancing the proinflammatory antipathogen immune response with the antiinflammatory response that limits tissue damage. We observed that almost all patients (nine of 10 cases) exhibiting the +3187 G-G genotype presented a severe form of leprosy, with a high bacillary load and multiple skin lesions; and all of them also carried the +3196 C allele. The only haplotype carrying both alleles associated with a more stable mRNA or less mRNA decay is UTR-1, that is, all these patients were homozygous for the UTR-1 haplotype, and UTR-1/UTR-1 homozygous has been implicated in high soluble HLA-G expression (Cristofaro et al. 2012). It was reported that T-cell activation induces HLA-G+ Treg cells to produce high levels of IL-10, which are responsible for T responder cell shift in cytokine expression from IFN-γ to IL-10-producing cells (Moraes et al. 2001; Walker and Lockwood 2006; Huang et al. 2009). It was also reported that a sustained leprosy antigen stimulus due to the deficient cell-mediated immunity in LL is responsible for the imbalance between T-effector and T_reg_ cells and the consequent Th2 immune phenotype observed in LL patients (Attia et al. 2010; Sampaio et al. 2012). Taken together, these observations suggest a possible role of HLA-G in the model of leprosy pathogenesis.

We also showed association of the high HLA-G producer UTR-1 haplotype with type I leprosy reaction, but no association with disease severity according to bacillary load (*P* = 0.223; OR = 1.45; 95% CI = 0.85–2.47). Type I leprosy reaction is observed in 50% of patients with MB leprosy and in almost all cases of reactive reaction in PB leprosy. Type I reaction is also characterized by delayed hypersensitivity reaction, in which T cell-mediated immunity drives the immune response to the tuberculoid pole of the leprosy spectrum (Cuevas et al. 2007). On the other hand, type II leprosy reaction has been reported to be associated with a significant decrease of T_reg_ cells, disease progression, and immune hyperactivity (Attia et al. 2010). The association of the +3187A allele and +3187 A-A genotype with type II reaction suggests that a decrease in HLA-G-expressing T cells may induce imbalance of the immune response to the presence of the mycobacterium-circulating antigen, favoring antigen–antibody complex deposit on vessel wall and tissue damage.

In conclusion, we showed that gene polymorphic sites associated with the modulation of the immunoregulatory HLA-G molecule are associated with polar forms of leprosy. 3′ UTR polymorphisms (+3187A-A and A-G genotypes) associated with low HLA-G expression were also associated with susceptibility to the development of PB leprosy. As a corollary, these polymorphisms are associated with protection against the development of the MB form. In the same context, the +3187A allele and the +3187A-A genotype were associated with type II reaction, a finding that is consistent with the low expression of HLA-G and exacerbation of the immune response, favoring an excessive production of circulating immune complex. Further studies evaluating HLA-G soluble levels should clarify the immunomodulatory role of HLA-G in the immune response in leprosy.
